# Supplementary material for: Prognostic significance of visit-to-visit variability, and maximum and minimum LDL cholesterol in diabetes mellitus
Source: Lipids Health Dis. 2022 Feb 10;21:19. doi: 10.1186/s12944-022-01628-8 (PMC8832816; doi:10.1186/s12944-022-01628-8)
Supplement: Supplementary file 1 — Additional file 1: [file 12944_2022_1628_MOESM1_ESM.pdf]

Prognostic significance of visit-to-visit variability, and maximum and minimum LDL cholesterol in diabetes mellitus

By Chang-Sheng Sheng

WORD COUNT 5553
TIME SUBMITTED 22-DEC-2021 01:54AM
PAPER ID 80566732

Prognostic significance of visit-to-visit variability, and maximum and minimum LDL cholesterol in diabetes mellitus

Short title: Prognostic significance of LDL-C variability

Chang-Sheng Sheng[1*], Ya Miao[2*], Lili Ding[3], Yi Cheng[1], Dan Wang[1], Yulin Yang[2], and Jingyan Tian[2]

[1]Department of Cardiovascular Medicine, Center for Epidemiological Studies and Clinical Trials and Center for Vascular Evaluation, Shanghai Key Lab of Hypertension, Shanghai Institute of Hypertension, Ruijin Hospital, Shanghai Jiaotong University School of Medicine, Shanghai, China

[2] State Key Laboratory of Medical Genomics, Clinical Trial Center, Shanghai Institute of Endocrine and Metabolic Diseases, Department of Endocrinology and Metabolism, Ruijin Hospital, Shanghai Jiaotong University School of Medicine, Shanghai, China

[3]Shanghai Key Laboratory of Complex Prescriptions and MOE Key Laboratory for Standardization of Chinese Medicines, Institute of Chinese Materia Medica, Shanghai University of Traditional Chinese Medicine, Shanghai, China.

[*]Chang-Sheng Sheng and Ya Miao contributed equally to this work.

Reprint requests and correspondence: Chang-Sheng Sheng and Jingyan Tian

Chang-Sheng Sheng, MD, PhD

Department of Cardiovascular Medicine, Center for Epidemiological Studies and Clinical Trials and Center for Vascular Evaluation, Shanghai Institute of Hypertension, Ruijin Hospital, Shanghai Jiaotong University School of Medicine, Shanghai, China. 197 Ruijin Er Road, Shanghai, 200025 China. Email: scsheng2004@163.com. Tel +86-21-64370045 ext 663203

Jingyan Tian, MD, PhD, State Key Laboratory of Medical Genomics, Clinical Trial Center, Shanghai Institute of Endocrine and Metabolic Diseases, Department of Endocrinology and Metabolism, Ruijin Hospital, Shanghai Jiaotong University School of Medicine, 197 Ruijin Er Road, Shanghai, 200025 China Email: tianjypaper@163.com

This work was supported by the Ministry of Health (2016YFC1300103 and 2016YFC0905001), the Chinese National Natural Science Foundation (81770418, 81400346 and 81270935), and Shanghai Pujiang Talents Plan (18PJ1407200).

Abstract

BACKGROUND: Current guidelines for dyslipidemia management recommend that the LDL-C goal be lower than 70 mg/dL. The present study investigated the prognostic significance of visit-to-visit variability in LDL-C, and minimum and maximum LDL-C during follow-up in diabetes mellitus.

METHODS: The risk of outcomes in relation to visit-to-visit LDL-C variability was investigated in the Action to Control Cardiovascular Risk in Diabetes (ACCORD) Lipid trial. LDL-C variability indices were coefficient of variation (CV), variability independent of the mean (VIM), and average real variability (ARV). Multivariable Cox proportional hazards models were employed to estimate the adjusted hazard ratio (HR) and 95% confidence interval (CI).

RESULTS: Compared with the placebo group (n=2667), the fenofibrate therapy group (n=2673) had a significantly (P<0.01) lower mean plasma triglyceride (152.5 *vs*. 178.6 mg/dL), and total cholesterol (158.3 vs.162.9 mg/dL) but a similar mean LDL-C during follow-up (88.2 *vs*. 88.6 mg/dL, P>0.05). All three variability indices were associated with primary outcome, total mortality and cardiovascular mortality both in the total population and in the fenofibrate therapy group but only with primary outcome in the placebo group. The minimum LDL-C but not the maximum during follow-up was significantly associated with various outcomes in the total population, fenofibrate therapy and placebo group. The minimum LDL-C during follow-up ≥70 mg/dL was associated with an increased risk for various outcomes.

CONCLUSIONS: Visit-to-visit variability in LDL-C was a strong predictor of outcomes, independent of mean LDL-C. Patients with LDL-C controlled to less than

70 mg/dL during follow-up might have a benign prognosis.

Key Words: LDL cholesterol, Variability, Diabetes mellitus, ACCORD Trial

ClinicalTrials.gov number: NCT 00000620.

Background

Increased low-density lipoprotein cholesterol (LDL-C) is an established risk factor for cardiovascular disease and events, and lipid-lowering therapy with statins has been proven to be an effective way to lower the risk of future cardiovascular events [1-3]. However, the role of monitoring the level of LDL-C using a target-oriental method in patients on lipid-lowering therapy remains controversial [4]. In addition, most observational studies or clinical trials focused only on the level of LDL-C initially or at the end of the study, and rarely on the variability or persistence of LDL-C throughout the trial process [5, 6].

Previous observational studies in diabetes have raised concerns on visit-to-visit lipid variability in relation to long-term major adverse cardiac events. The post-hoc analysis of the Treating to New Targets (TNT) trial showed that visit-to-visit LDL-C variability was an independent predictor of cardiovascular events in patients 35 to 75 years of age who had known coronary artery disease [7]. However, no studies have concerned the prognostic value of visit-to-visit LDL-C variability and persistence of LDL-C control in type 2 diabetes at high cardiovascular risk.

Recent Joint European Society of Cardiology (ESC)/European Atherosclerosis Society (EAS) dyslipidemia guidelines recommended that LDL-C levels should be lowered as much as possible to prevent cardiovascular disease, especially in high and very high-risk patients [8]. In high-risk patients, such as general diabetes mellitus, the LDL-C goal is <70 mg/dL or at least 50% reduction from baseline LDL-C levels. Thus, the benefits of persistence of LDL-C controlled to below 70 mg/dL might be a hot topic.

In the present study, we employed data from the Action to Control Cardiovascular Risk in Diabetes (ACCORD) Lipid trial to investigate the associations between

visit-to-visit variability in LDL-C and primary outcome, and total and cardiovascular mortality in patients with type 2 diabetes who were at high risk for cardiovascular disease [9]. The purpose of the present study was to investigate the prognostic significance of and visit-to-visit variability, and maximum and minimum LDL-C in diabetes mellitus already receiving lipid-lowering drugs, beyond the mean levels of LDL-C.

METHODS

Study Population

The ACCORD study was conducted at 77 clinical sites in the United States and Canada. The rationale, design, inclusion criteria, subject characteristics, and main results of the ACCORD trial have been described (online study protocols: https://biolincc.nhlbi.nih.gov/studies/accord/) [9-13]. In brief, the participants were aged between 40 and 79 years, had type 2 diabetes mellitus and a glycated hemoglobin level of ≥7.5%, had previous evidence of clinical cardiovascular disease or at least two additional risk factors, and did not have a history of frequent or recent serious hypoglycemic events. All patients were randomly assigned to receive either intensive glycemic control targeting a glycated hemoglobin level below 6.0% or standard therapy targeting a glycated hemoglobin level of 7.0 to 7.9%.

The ACCORD Lipid trial was conducted in a subgroup of patients in the ACCORD study, and was also randomized in a 2-by-2 factorial design. Open-label simvastatin treatment started at the randomization time and either fenofibrate or placebo was masked one month later. Randomization occurred between January 11, 2001, and October 29, 2005. End-of-study visits were scheduled between March and June 2009.

Patients were specifically eligible to participate in the lipid trial if they also had the

following: an LDL cholesterol level of 60 to 180 mg/dL, an HDL-C (high-density lipoprotein cholesterol) level below 55 mg/dL for women and blacks or below 50 mg/dL for all other groups, and a triglyceride level below 750 mg/dL if they were not receiving lipid therapy or below 400 mg/dL if they were receiving lipid therapy. The exclusion criteria included the use of a drug known to interact with statins or fibrate; history of pancreatitis, myositis/myopathy, or gallbladder disease; or refusal to stop any current lipid-altering treatment. All patients provided written informed consent.

Data acquisition

A fasting plasma lipid profile was measured at the ACCORD central laboratory at 4, 8, and 12 months after randomization, annually thereafter, and at the end of the study.

The primary outcome was the first occurrence of a major cardiovascular event, including nonfatal myocardial infarction, nonfatal stroke, or death from cardiovascular causes. The total and cardiovascular mortality was death from any cause and from cardiovascular causes, respectively.

Data Analysis

SAS software (Version 9.4, SAS Institute Inc, Cary, NC) was used for database management and statistical analysis. Means and proportions were compared using the large-sample z test and the $\chi 2$ statistic, respectively. Characteristics of the study population included in the present analyses were shown by therapy status (fenofibrate vs placebo) and baseline LDL variability levels.

The visit-to-visit LDL-C variability was evaluated using at least 3 measurements from the beginning to the end of the study, and individual coefficient of variation (CV), independent of the mean (VIM) [14], and average real variability (ARV) [15] were calculated. CV was calculated as the standard deviation (SD) divided by the

mean. VIM was calculated as the SD divided by the mean to the power x and multiplied by the population mean to the power x, with x derived from curve fitting. VIM can diminish the tight correlation between the CV and mean. ARV was calculated as the average of the absolute differences between consecutive LDL-C measurements. To study the association between outcomes and LDL-C variability, we first searched for covariables associated with LDL-C variability in stepwise regression analysis with P values for explanatory variables to enter and stay in models set at 0.15. The prognostic significance of LDL-C variability for various outcomes was determined in multivariable Cox proportional hazards models, while adjusting for sex, therapy group, and baseline age, education, body mass index, systolic and diastolic blood pressure, and fasting plasma glucose. Two models were conducted as if it was additionally adjusted for the mean LDL-C during visits or not.

The variability and maximum and minimum LDL-C were investigated as continuous variables using Cox proportional hazards models, and the hazard ratios (HRs) for various outcomes of one SD increment in LDL-C variability indices were reported. The maximum and minimum LDL_C was also investigated as a categorical variable and the HRs for various outcomes of ≥70 vs. <70 mg/dL were reported. In addition, HRs and 95% confidence intervals (CIs) for each decile relative to the first decile in the placebo group and for each 10-percentile point increase in variability were estimated in a single model. Significance was a 2-tailed α-level of ≤0.05.

RESULTS

Characteristics of the Study Participants

Of all 5518 participants, 5340 unerwent LDL-C measurement on at least 3 visits during the study and were included in this analysis. The 5340 participants included

1632 women (30.6%) and had a mean age of 62.8 (±6.6) years old. Key baseline characteristics were similar in the two therapy groups (**Table 1**).

Compared with the placebo group, the fenofibrate group had significantly (P<0.001) lower total cholesterol (158.3 *vs.* 162.9 mg/dL) and triglyceride levels (152.5 *vs.* 178.6 mg/dL), but higher HDL levels (40.2 vs. 39.5 mg/dl). For LDL-C levels, the fenofibrate group showed similar mean LDL-C and higher maximum LDL-C (120.1 vs. 119.0 mg/dL but lower minimum LDL-C (63.7 vs. 64.7 mg/dL). For LDL-C variability indices, the fenofibrate group showed no difference in mean LDL-C level and LDL-C VIM but lower SD and ARV (all P<0.001, **Table 1 and Figure 1A)**.

Compared with the low LDL-C variability (VIM<13.2) group, the high LDL-C variability (VIM≥13.2) group had significantly greater baseline body weight and waist circumference, and significantly (P<0.0001) higher baseline systolic and diastolic blood pressure, fasting serum glucose, and total, HDL and LDL cholesterol, but lower triglyceride levels. The increased LDL-C variability group had significantly (P<0.0001) higher total and LDL-C and triglyceride, but lower HDL cholesterol. As expected, the increased LDL-C variability group had significantly (P<0.0001) higher various LDL-C variability indices, including SD, VIM, and ARV (**Table 1**).

Variability Indices and Outcomes

During the trial, the primary outcome, all-cause deaths and cardiovascular deaths occurred in 276, 179 and 87 subjects in the fenofibrate group, respectively, and in 294, 201 and 102 subjects in the placebo group, respectively. In multiple Cox regression analyses adjusted for sex and age, education, waist circumference, body mass index, systolic and diastolic blood pressure, and fasting plasma glucose at baseline, and additionally mean LDL-C during follow-up, all three LDL-C variability indices were significantly (P<0.001) associated with primary outcome, and all-cause and

cardiovascular deaths in total population and the Fenofibrate group. However, in the placebo group, only LDL-C ARV was significantly associated with total and cardiovascular deaths (**Table 2**).

To allow for nonlinearity, all three LDL-C variability indices were split into deciles and HRs were calculated in relation to the first decile in the placebo group. For the primary outcome, only the 10th decile of LDL-C VIM and ARV in both groups had a significantly higher risk (**Figure 1B**). For all-cause deaths, only the 10th decile of LDL-C CV in the intensive-therapy group had marginally significantly higher risk (**Figure 1C**). For cardiovascular deaths, some deciles of LDL-C variability indices had significantly lower risk but not higher risk (**Figure 1D**).

Maximum and Minimum LDL_C during Follow-Up and Outcomes

In multiple Cox regression analyses, the mean LDL-C during follow-up was significantly associated with primary outcome, total mortality, and cardiovascular mortality in the total population, the fenofibrate group, and the placebo group (**Table 2**). The prognostic variability of maximum and minimum LDL-C during follow-up was further investigated to look at the most benefits of lipid control. In multivariate analysis adjusted for other covariates and mean LDL-C during follow-up, the minimum but not the maximum LDL-C was more frequently significantly associated with the primary outcome, and total and cardiovascular deaths in the total population, as well as in the fenofibrate and placebo groups analyzed separately. The hazard ratios of the 1-SD increase in minimum LDL-C were 1.54 (95%CI, 1.41-1.67), 1.41 (1.28-1.56), and 1.54 (1.34-1.77) for the primary outcome, all-cause deaths and cardiovascular deaths, respectively in the total population (**Table 3**).

Further, the maximum and minimum LDL-C exceed 70 mg/dl, the threshold recommended by recent guideline, was investigated in relation to various outcomes.

In a similar adjusted analysis, the minimum but not the maximum LDL-C exceeding 70 mg/dL was significantly (*P*≤0.01) associated with the primary outcome, and total and cardiovascular deaths in the total population, as well as in the fenofibrate and placebo groups analyzed separately. The hazard ratios of minimum LDL-C≥ 70 mg/dL were 2.11 (95%CI, 1.77-2.52), 1.60 (1.31-1.97), and 2.03 (1.52-2.70) for the primary outcome, all-cause deaths and cardiovascular deaths, respectively in the total population (**Table 3**).

Discussion

In the present study, three variability indices of LDL-C (CV, VIM and ARV) were analyzed in type 2 diabetes. The key findings can be summarized in 3 points: (1) visit-to-visit variability in LDL-C was an independent and powerful predictor of primary outcome, all-cause and cardiovascular deaths, independent of mean LDL-C and Fenofibrate treatment effect; (2) the minimum but not the maximum LDL-C were significantly associated with the various outcomes in both the Fenofibrate and placebo groups; (3) the minimum LDL-C exceed 70 mg/dL, the threshold recommended by recent guideline, was associated with various outcomes. These findings raised the issue that visit-to-visit LDL-C variability might be an important risk factor for outcomes, and LDL-C able to control to less than 70 mg/dL at least once might have a benign prognosis.

Several observational studies confirmed the relationship between LDL-C variability and major adverse cardiac events in patients after ST-segment elevation myocardial infarction [16], patients with previous myocardial infarction [17], or elderly patients at high risk of vascular disease [18]. Analysis from the TNT (Treating to New Targets) trial showed that visit-to-visit LDL-C variability is an independent predictor of cardiovascular events in subjects with coronary artery disease [19]. In this study, a

1-SD increase in LDL-C variability conferred a 10% to 23% higher risk of any coronary event, any cardiovascular event, death, myocardial infarction, and stroke. The results of the present study also indicate that visit-to-visit variability in LDL-C (SD, CV, and ARV) was lower in the fenofibrate group than in the placebo group, irrespective of the statin therapy status. However, the mean LDL-C level was similar between the two groups, which showed that fenofibrate did not reduce LDL_C levels but did reduce LDL-C variability. LDL-C variability (CV and VIM) was only significantly associated with both total and cardiovascular mortality in the fenofibrate group but not in the placebo group. However, the ARV of LDL-C was associated with various outcomes in both the fenofibrate and placebo groups.

To the best of our knowledge, the current analysis was the first to study the prognostic significance for LDL-C variability in type 2 diabetes. In 864 patients with type 2 diabetes aged 62.7 (±11.8) years, with a median follow-up of 3.8 years, HDL-C rather than LDL-C variability was associated with a higher risk of diabetic nephropathy progression [20]. Another study investigated the association between the variability of LDL-C, systolic blood pressure, diastolic blood pressure, and total-, HDL- and LDL-cholesterol in type 2 diabetic patients with the risk of diabetic kidney disease [21]. The study found that the combination of high variability in LDL-C and HDL-C conferred the highest risk of developing albuminuria (HR 1.47; 95% CI 1.17-1.84). The present study confirmed that high LDL-C variability was a predictor of primary outcome and mortality in diabetes mellitus.

The exact mechanism concerning increased LDL-C variability to a high risk of primary outcome and total and cardiovascular deaths remains unknown. However, there are several possible explanations. Because greater LDL-C variability might increase the likelihood of plaque vulnerability and rupture, it may lead to instability at

the vascular wall, as a result of variability in lipid efflux mechanisms, thereby increasing the risk of cardiovascular events [19]. Under the conditions of high plasma glucose or diabetes mellitus, the detriment of atherosclerosis might be amplified. In fact, associations between diabetes and atherosclerosis are well established [22]). Numerous data from clinical trials and experimental experiments showing the onset of diabetes mellitus complications are associated with atherosclerosis, which means that the important role of diabetes mellites might induce damage on endothelial function, and then cause instability in vascular homeostasis [23, 24].

Recent dyslipidemia management guidelines recommended that LDL-C levels should be lowered as much as possible to prevent cardiovascular disease, especially in high and very high-risk patients. In high-risk patients, such as general diabetes mellitus, the LDL-C goal is <70 mg/dL or at least 50% reduction from baseline LDL-C levels [8]. The present study was the first to investigate the prognostic significance of minimum and maximum LDL-C during follow-up, and found that the minimum but not the maximum LDL-C was significantly associated with the primary outcome, and total and cardiovascular deaths in both the fenofibrate and placebo groups and the minimum LDL-C exceeding 70 mg/dL were associated with various outcomes. The results mean that LDL-C able to be controlled to less than 70 mg/dL during follow-up might have a benign prognosis.

Strengths and limitations

The present study should be interpreted within the context of its strengths and limitations. The main strengths of this study include a large number of LDL-C measures, which enable us to accurately calculate LDL-C variability. In addition, as many as three variability indices were used, which enabled us to study LDL-C variability more comprehensively. Furthermore, for primary outcome analysis, we

calculated time-dependent measures of variation before the events occurred. The analyses also have limitations. Because of the post hoc nature of the analysis and the highly selected study population, the results should be investigated in other studies and extended to real world studies. Another limitation was that this study was a fenofibrate rather than statin treatment trial, and information on statin usage, which affecst the stability of LDL-C, was lacking. The ACCORD study enrolled patients more than a decade ago (2001-2009). The study results might be therefore not applicable to contemporary real-world patients. However, the prevalence of dyslipidemia and other risk factors in this population is similar to that of contemporary trials [25] and real-world registries [26], and therefore can be generalized of the results in more contemporary cohorts.

Perspectives

Visit-to-visit variability in LDL-C was a strong predictor of outcomes, and an LDL-C was control to less than 70 mg/dL might have a benign prognosis. The present study implies that an LDL-C goal of long-term low level during lipid therapy confers the largest benefit in patients with high cardiovascular risk. In recent years, combining PCSK9 inhibitors with statins and/or ezetimibe allowed to substantially reduce LDL-C levels, improve the control of LDL-C levels over time, increase treatment adherence, and might therefore reduce the LDL-C variability，with relevant effects on cardiovascular outcomes[27, 28].

Acknowledgements The investigators acknowledge and thank the ACCORD investigators and the National Heart, Lung, and Blood Institute for conducting the trials and making datasets publicly available.

Authors' contributions All authors participated in critical revision of the manuscript for important intellectual content. C.-S.S. and Y.M. contributed to the statistical analysis and wrote the manuscript. C.-S.S., Y.M., L.D., Y.C., D.W.; and Y.Y. participated in the acquisition, analysis, or interpretation of data. C.-S.S., and J.T. reviewed and edited the manuscript. C.-S.S., and J.T. is the guarantor of the work and as such, had full access to all the data in the study and takes responsibility for the integrity of the data and the accuracy of the data analysis.

Funding This work was supported by the Ministry of Health (2016YFC1300103 and 2016YFC0905001), the Chinese National Natural Science Foundation (81770418, 81400346 and 81270935), and Shanghai Pujiang Talents Plan (18PJ1407200).

Availability of data and materials

The datasets used and/or analysed during the current study are available from the corresponding author on reasonable request or the ACCORD trial group.

Disclosures of Interest No potential conflicts of interest relevant to this article were reported.

Ethics approval and consent to participate

The protocol was approved by the institutional review board or ethics committee at each center and by an independent protocol review committee appointed by the NHLBI.

Declaration of Helsinki The authors state that this study complies with the

Declaration of Helsinki.

Reference

1. Cholesterol Treatment Trialists C, Baigent C, Blackwell L, Emberson J, Holland LE, Reith C, Bhala N, et al. Efficacy and safety of more intensive lowering of LDL cholesterol: a meta-analysis of data from 170,000 participants in 26 randomised trials. Lancet 2010;**376**(9753):1670-81.
2. Baigent C, Keech A, Kearney PM, Blackwell L, Buck G, Pollicino C, et al, Cholesterol Treatment Trialists C. Efficacy and safety of cholesterol-lowering treatment: prospective meta-analysis of data from 90,056 participants in 14 randomised trials of statins. Lancet 2005;**366**(9493):1267-78.
3. Cholesterol Treatment Trialists C. Efficacy and safety of statin therapy in older people: a meta-analysis of individual participant data from 28 randomised controlled trials. Lancet 2019;**393**(10170):407-415.
4. Stone NJ, Robinson JG, Lichtenstein AH, Bairey Merz CN, Blum CB, Eckel RH, et al, American College of Cardiology/American Heart Association Task Force on Practice G. 2013 ACC/AHA guideline on the treatment of blood cholesterol to reduce atherosclerotic cardiovascular risk in adults: a report of the American College of Cardiology/American Heart Association Task Force on Practice Guidelines. Circulation 2014;**129**(25 Suppl 2):S1-45.
5. Sabatine MS, De Ferrari GM, Giugliano RP, Huber K, Lewis BS, Ferreira J, et al. Clinical Benefit of Evolocumab by Severity and Extent of Coronary Artery Disease: Analysis From FOURIER. Circulation 2018;**138**(8):756-766.
6. Ray KK, Colhoun HM, Szarek M, Baccara-Dinet M, Bhatt DL, Bittner VA, et al, Committees OO, Investigators. Effects of alirocumab on cardiovascular and metabolic outcomes after acute coronary syndrome in patients with or without diabetes: a prespecified analysis of the ODYSSEY OUTCOMES randomised controlled trial. Lancet Diabetes Endocrinol 2019;**7**(8):618-628.
7. Waters DD, Bangalore S, Fayyad R, DeMicco DA, Laskey R, Melamed S, et al. Visit-to-visit variability of lipid measurements as predictors of cardiovascular events. J Clin Lipidol 2018;**12**(2):356-366.
8. Mach F, Baigent C, Catapano AL, Koskinas KC, Casula M, Badimon L, et al, Group ESCSD. 2019 ESC/EAS Guidelines for the management of dyslipidaemias: lipid modification to reduce cardiovascular risk. Eur Heart J 2020;**41**(1):111-188.
9. Group AS, Ginsberg HN, Elam MB, Lovato LC, Crouse JR, Leiter LA, et al. Effects of combination lipid therapy in type 2 diabetes mellitus. N Engl J Med 2010;**362**(17):1563-74.

10. Elam MB, Ginsberg HN, Lovato LC, Corson M, Largay J, Leiter LA, et al. Association of Fenofibrate Therapy With Long-term Cardiovascular Risk in Statin-Treated Patients With Type 2 Diabetes. JAMA Cardiol 2017;**2**(4):370-380.
11. Bonds DE, Craven TE, Buse J, Crouse JR, Cuddihy R, Elam M, et al. Fenofibrate-associated changes in renal function and relationship to clinical outcomes among individuals with type 2 diabetes: the Action to Control Cardiovascular Risk in Diabetes (ACCORD) experience. Diabetologia 2012;**55**(6):1641-50.
12. Frazier R, Mehta R, Cai X, Lee J, Napoli S, Craven T, et al. Associations of Fenofibrate Therapy With Incidence and Progression of CKD in Patients With Type 2 Diabetes. Kidney Int Rep 2019;**4**(1):94-102.
13. Ginsberg HN, Bonds DE, Lovato LC, Crouse JR, Elam MB, Linz PE, et al. Evolution of the lipid trial protocol of the Action to Control Cardiovascular Risk in Diabetes (ACCORD) trial. Am J Cardiol 2007;**99**(12A):56i-67i.
14. Rothwell PM, Howard SC, Dolan E, O'Brien E, Dobson JE, Dahlof B, et al. Prognostic significance of visit-to-visit variability, maximum systolic blood pressure, and episodic hypertension. Lancet 2010;**375**(9718):895-905.
15. Mena L, Pintos S, Queipo NV, Aizpurua JA, Maestre G, Sulbaran T. A reliable index for the prognostic significance of blood pressure variability. J Hypertens 2005;**23**(3):505-11.
16. Boey E, Gay GM, Poh KK, Yeo TC, Tan HC, Lee CH. Visit-to-visit variability in LDL- and HDL-cholesterol is associated with adverse events after ST-segment elevation myocardial infarction: A 5-year follow-up study. Atherosclerosis 2016;**244**:86-92.
17. Bangalore S, Fayyad R, Messerli FH, Laskey R, DeMicco DA, Kastelein JJ, et al. Relation of Variability of Low-Density Lipoprotein Cholesterol and Blood Pressure to Events in Patients With Previous Myocardial Infarction from the IDEAL Trial. Am J Cardiol 2017;**119**(3):379-387.
18. Smit RA, Trompet S, Sabayan B, le Cessie S, van der Grond J, van Buchem MA, et al. Higher Visit-to-Visit Low-Density Lipoprotein Cholesterol Variability Is Associated With Lower Cognitive Performance, Lower Cerebral Blood Flow, and Greater White Matter Hyperintensity Load in Older Subjects. Circulation 2016;**134**(3):212-21.
19. Bangalore S, Breazna A, DeMicco DA, Wun CC, Messerli FH, Committee TNTS, Investigators. Visit-to-visit low-density lipoprotein cholesterol variability and risk of cardiovascular outcomes:

insights from the TNT trial. J Am Coll Cardiol 2015;**65**(15):1539-48.

20. Chang YH, Chang DM, Lin KC, Hsieh CH, Lee YJ. High-density lipoprotein cholesterol and the risk of nephropathy in type 2 diabetic patients. Nutr Metab Cardiovasc Dis 2013;**23**(8):751-7.

21. Becker RHA. In response to: Heise T, Norskov M, Nosek L, Kaplan K, Famulla S and Haahr H. L. (2017) Insulin degludec: Lower day-to-day and within-day variability in pharmacodynamic response compared to insulin glargine U300 in type 1 diabetes. Diabetes Obes Metab. 2017;19:1032-1039. Diabetes Obes Metab 2018;**20**(8):2043-2047.

22. Poznyak A, Grechko AV, Poggio P, Myasoedova VA, Alfieri V, Orekhov AN. The Diabetes Mellitus-Atherosclerosis Connection: The Role of Lipid and Glucose Metabolism and Chronic Inflammation. Int J Mol Sci. 2020;21(5):1835.

23. Kaur R, Kaur M, Singh J. Endothelial dysfunction and platelet hyperactivity in type 2 diabetes mellitus: molecular insights and therapeutic strategies. Cardiovasc Diabetol. 2018;17(1):121.

24. Iwakawa N, Tanaka A, Ishii H, Kataoka T, Niwa K, Hitora Y, Tashiro H, Mitsuda T, Kojima H, Hirayama K, Furusawa K, Yoshida R, Suzuki S, Murohara T. Impact of Diabetes Mellitus on the Aortic Wall Changes as Atherosclerosis Progresses: Aortic Dilatation and Calcification. J Atheroscler Thromb. 2020;27(6):509-515.

25. Valgimigli M, Gragnano F, Branca M, Franzone A, Baber U, Jang Y, et al. P2Y12 inhibitor monotherapy or dual antiplatelet therapy after coronary revascularisation: individual patient level meta-analysis of randomised controlled trials. BMJ. 2021; 373:n1332

26. Cesaro A, Gragnano F, Calabrò P, Moscarella E, Santelli F, Fimiani F, et al. Prevalence and clinical implications of eligibility criteria for prolonged dual antithrombotic therapy in patients with PEGASUS and COMPASS phenotypes: Insights from the START-ANTIPLATELET registry. Int J Cardiol. 2021 Dec 15;345:7-13.

27. Gragnano F, Natale F, Concilio C, Fimiani F, Cesaro A, Sperlongano S ,et al. Adherence to proprotein convertase subtilisin/kexin 9 inhibitors in high cardiovascular risk patients: an Italian single-center experience J Cardiovasc Med (Hagerstown). 2018;19(2):75-77.

28. Cesaro A, Gragnano F, Fimiani F, Moscarella E, Diana V, Pariggiano I, et al. Impact of PCSK9 inhibitors on the quality of life of patients at high cardiovascular risk. Eur J Prev Cardiol. 2020;27(5):556-558.

Figure Legend

Figure . Hazard ratios for risk of outcomes by decile of LDL cholesterol variability indices. All hazard ratios for the primary outcome (B), all-cause death (C) and cardiovascular death (D) were adjusted for the mean lipid during visits, sex, and baseline age, education, body mass index, systolic and diastolic blood pressure, smoking, drinking, and fasting plasma glucose. Hazard ratios and 95% confidence intervals for each decile relative to the first decile in the placebo group and for each 10-percentile point increase in variability were estimated in a single model. The distributions of variability indices are also shown (A). VIM indicates variability independent of the mean (left); ARV, average real variability (middle); and MMD, the difference of maximum minus minimum LDL_C (right).

Table 1. Characteristics of the patients at baseline or during follow-up

	All patients	Therapy Status[a]		LDL variability[b]		
	(n=5340)	Fenofibrate (n=2673)	Placebo (n=2667)	VIM <13.2 (n=2665)	VIM ≥13.2 (n=2675)	*P*
**At baseline**						
Age	62.8±6.6	62.8±6.5	62.8±6.7	62.9±6.6	62.6±6.5	0.06
Female sex (n, %)	1632 (30.6)	817 (30.6)	815 (30.6)	770 (28.9)	862 (32.2)	0.008
Weight	94.9±18.3	94.6±18.2	95.2±18.5	95.6±17.9	94.3±18.7	0.009
Body-mass index	32.3±5.3	32.2±5.3	32.4±5.3	32.4±5.3	32.2±5.4	0.28
Waist circumference (cm)	107.7±13.5	107.5±13.3	107.8±13.7	108.1±13.4	107.2±13.6	0.02
Systolic blood pressure (mmHg)	133.9±17.7	133.8±17.6	133.9±17.9	132.2±17.2	135.5±18.1	<0.0001
Diastolic blood pressure (mmHg)	74.0±10.8	73.8±10.6	74.1±10.9	73.1±10.6	74.8±10.9	<0.0001
Fasting serum glucose (mg/dL)	175.8±54.6	176.3±54.1	175.3±55.1	170.7±51.8	180.8±56.8	<0.0001
Total cholesterol (mg/dL)	175.3±37.4	174.9±36.8	175.7±38.0	162.8±28.2	187.8±41.1	<0.0001
LDL cholesterol (mg/dL)	100.6±30.7	100.0±30.2	101.2±31.0	91.5±24	109.6±33.8	<0.0001
HDL cholesterol (mg/dL)	38.1±7.8	38.0±7.8	38.2±7.7	38.5±7.7	37.7±7.8	0.0005
Plasma triglyceride (mg/dL)	188.0±113	189.7±111,5	186.3±114.6	167±91.1	208.9±127.9	<0.0001
Serum creatinine (mg/dL)	0.92±0.22	0.93±0.23	0.93±0.22	0.92±0.23	0.93±0.21	0.04
**Lipids during follow-up**						
Mean total cholesterol (mg/dL)	160.6±26.8	158.3±26.2	162.9±27.2*	156.9±23.6	164.3±29.1	<0.0001
Mean plasma triglyceride (mg/dL)	165.5±88.5	152.5±80.6	178.6±94.0*	151.4±72.6	179.5±100	<0.0001
Mean HDL cholesterol (mg/dL)	39.9±8.2	40.2±8.7	39.5±7.8*	40.5±8.3	39.2±8.1	<0.0001
Mean LDL cholesterol (mg/dL)	88.4±19.8	88.2±19.9	88.6±19.7	86.6±18.4	90.1±21.0	<0.0001

Maximum LDL cholesterol (mg/dL)	120.1±29.6	119.0±29.6	121.3±29.5*	107.4±22.7	132.8±30.2	<0.0001
Minimum LDL cholesterol (mg/dL)	64.2±17.8	64.7±17.5	63.7±18.0‡	68.2±17.1	60.1±17.5	<0.0001
LDL SD	19.3±8.8	18.7±8.8	19.9±8.8*	13.5±4.8	25.1±8.1	<0.0001
LDL CV (%)	22.1±9.2	21.4±9.0	22.7±9.4*	15.8±5.3	28.3±8.0	<0.0001
LDL VIM	13.9±6.0	13.8±6.0	14.1±5.9	9.3±2.5	18.5±4.8	<0.0001
LDL ARV	18.4±9.6	17.7±9.5	19.0±9.6*	13.7±5.8	23.0±10.3	<0.0001

Values were means (SD) or median (quartile). VIM indicates variability independent of the mean; ARV, average real variability; and MMD, the difference of maximum minus minimum LDL. [a]The Fenofibrate vs Placebo group, *$P<0.001$; †$P<0.01$; and ‡$P<0.05$. [b]High vs Low VIM, and the P value is given.

Table 2 Association of mean and variability indexes of LDL cholesterol during follow-up with outcomes

Outcomes	Model	Total population (n=5340)		Fenofibrate（n=2673）		Placebo (n=2667)	
		HR (95%CI)	*P*	HR (95%CI)	*P*	HR (95%CI)	*P*
**Primary outcome**							
Mean (+20 mg/dL)	Model 1	1.32 (1.16-1.33)	0.001	1.31 (1.19-1.45)	<0.0001	1.34 (1.16-1.54)	<0.0001
CV (+9.2%)	Model 1	1.19 (1.10-1.29)	<0.0001	1.31 (1.17-1.46)	<0.0001	1.09 (0.97-1.22)	0.13
	Model 2	1.22 (1.13-1.32)	<0.0001	1.33 (1.19-1.48)	<0.0001	1.12 (1.00-1.26)	0.04
VIM (+6 U)	Model 1	1.30 (1.21-1.39)	<0.0001	1.33 (1.22-1.46)	<0.0001	1.26 (1.13-1.40)	<0.0001
	Model 2	1.29 (1.20-1.38)	<0.0001	1.33 (1.21-1.45)	<0.0001	1.25 (1.12-1.39)	<0.0001
ARV (+10 mg/dL)	Model 1	1.28 (1.19-1.38)	<0.0001	1.30 (1.18-1.43)	<0.0001	1.24 (1.11-1.39)	<0.0001
	Model 2	1.27 (1.17-1.38)	<0.0001	1.33 (1.19-1.49)	<0.0001	1.19 (1.05-1.35)	0.005
**Total mortality**							
Mean (+20 mg/dL)	Model 1	1.32 (1.19-1.46)	<0.0001	1.25 (1.07-1.46)	0.004	1.37 (1.20-1.56)	<0.0001
CV (+9.2%)	Model 1	1.07 (0.97-1.18)	0.19	1.26 (1.10-1.44)	0.001	0.93 (0.81-1.07)	0.29
	Model 2	1.12 (1.01-1.23)	0.03	1.29 (1.13-1.49)	0.0003	0.98 (0.85-1.13)	0.77
VIM (+6 U)	Model 1	1.15 (1.05-1.27)	0.004	1.26 (1.12-1.43)	0.0003	1.06 (0.92-1.22)	0.45
	Model 2	1.13 (1.03-1.25)	0.01	1.25 (1.10-1.42)	0.0007	1.04 (0.90-1.19)	0.63
ARV (+10 mg/dL)	Model 1	1.37 (1.26-1.49)	<0.0001	1.35 (1.21-1.52)	<0.0001	1.40 (1.24-1.59)	<0.0001
	Model 2	1.29 (1.17-1.42)	<0.0001	1.31 (1.15-1.51)	<0.0001	1.29 (1.12-1.48)	0.0006
**Cardiovascular mortality**							
Mean (+20 mg/dL)	Model 1	1.34 (1.16-1.54)	<0.0001	1.30 (1.05-1.62)	0.018	1.36 (1.13-1.64)	0.001
CV (+9.2%)	Model 1	1.04 (0.90-1.20)	0.59	1.33 (1.10-1.61)	0.003	0.83 (0.67-1.01)	0.07
	Model 2	1.09 (0.94-1.26)	0.27	1.37 (1.13-1.66)	0.001	0.86 (0.70-1.07)	0.17

VIM (+6 U)	Model 1	1.17 (1.02-1.34)	0.02	1.38 (1.18-1.61)	<0.0001	0.96 (0.78-1.18)	0.68
	Model 2	1.15 (1.00-1.31)	0.047	1.35 (1.16-1.59)	0.0002	0.94 (0.76-1.15)	0.53
ARV (+10 mg/dL)	Model 1	1.37 (1.22-1.55)	<0.0001	1.44 (1.24-1.68)	<0.0001	1.29 (1.07-1.55)	0.008
	Model 2	1.28 (1.11-1.47)	0.0006	1.40 (1.17-1.68)	0.0003	1.15 (0.93-1.42)	0.19

Model 1 was adjusted for therapy group (if applicable), sex, and baseline age, education, body mass index, systolic and diastolic blood pressure, smoking, drinking, and fasting plasma glucose. Model 2 was additionally for mean LDL-C during visits.. VIM indicates variability independent of the mean; ARV, average real variability; and MMD, the difference of maximum minus minimum LDL-C.

Table 3. Hazard ratios for top decile of maximum and minimum LDL cholesterol during follow-up for Outcomes

	Total population (n=5340)		Fenofibrate（n=2673）		Placebo (n=2667)	
	**HR (95%CI)**	***P***	**HR (95%CI)**	***P***	**HR (95%CI)**	***P***
Maximum LDL_C						
+1 SD (30 mg/dL)						
Primary outcome	1.10 (1.01-1.20)	0.04	1.15 (1.01-1.31)	0.03	1.15 (1.01-1.31)	0.03
All-cause deaths	1.12 (1.01-1.23)	0.04	1.16 (0.99-1.34)	0.06	1.08 (0.94-1.24)	0.30
Cardiovascular deaths	1.15 (0.99-1.33)	0.054	1.26 (1.02-1.56)	0.03	1.05 (0.87-1.28)	0.60
≥70 **vs. <70** mg/dL						
Primary outcome	0.85 (0.48-1.52)	0.59	0.75 (0.33-1.69)	0.49	0.98 (0.43-2.20)	0.95
All-cause deaths	0.61 (0.34-1.12)	0.11	0.50 (0.21-1.24)	0.13	0.70 (0.31-1.61)	0.40
Cardiovascular deaths	0.48 (0.23-1.03)	0.06	0.62 (0.15-2.56)	0.51	0.43 (0.17-1.07)	0.07
Minimum LDL_C						
+1 SD (18 mg/dL)						
Primary outcome	1.54 (1.41-1.67)	<0.0001	1.48 (1.31-1.68)	<0.0001	1.60 (1.42-1.80)	<0.0001
All-cause deaths	1.41 (1.28-1.56)	<0.0001	1.31 (1.13-1.53)	0.0005	1.50 (1.31-1.70)	<0.0001
Cardiovascular deaths	1.54 (1.34-1.77)	<0.0001	1.43 (1.15-1.78)	0.001	1.63 (1.36-1.96)	<0.0001
≥70 *vs*. <70 mg/dL						
Primary outcome	2.11 (1.77-2.52)	<0.0001	1.95 (1.51-2.52)	<0.0001	2.30 (1.79-2.94)	<0.0001
All-cause deaths	1.60 (1.31-1.97)	<0.0001	1.48 (1.10-1.99)	0.01	1.73 (1.30-2.28)	0.0001
Cardiovascular deaths	2.03 (1.52-2.70)	<0.0001	1.70 (1.11-2.60)	0.02	2.39 (1.61-3.53)	<0.0001

All models were adjusted for therapy group (if applicable), sex, and baseline age, education, body mass index, systolic and diastolic blood pressure, smoking, drinking, and fasting plasma glucose.

Prognostic significance of visit-to-visit variability, and maximum and minimum LDL cholesterol in diabetes mellitus

ORIGINALITY REPORT

73%

SIMILARITY INDEX

MATCH ALL SOURCES (ONLY SELECTED SOURCE PRINTED)

★Chang-Sheng Sheng, Ya Miao, Lili Ding, Yi Cheng, Dan Wang, Yuling Yang, Jingyan Tian. "Prognostic Significance of Visit-To-Visit Variability, Maximum and Minimum LDL Cholesterol in Diabetes Mellitus", Research Square Platform LLC, 2021 — 67%

Crossref Posted Content

EXCLUDE QUOTES	OFF	EXCLUDE MATCHES	OFF
EXCLUDE BIBLIOGRAPHY	OFF		
