# Supplementary figures and images for: Prognostic significance of visit-to-visit variability, and maximum and minimum LDL cholesterol in diabetes mellitus
Source: Lipids Health Dis. 2022 Feb 10;21:19. doi: 10.1186/s12944-022-01628-8 (PMC8832816; doi:10.1186/s12944-022-01628-8)

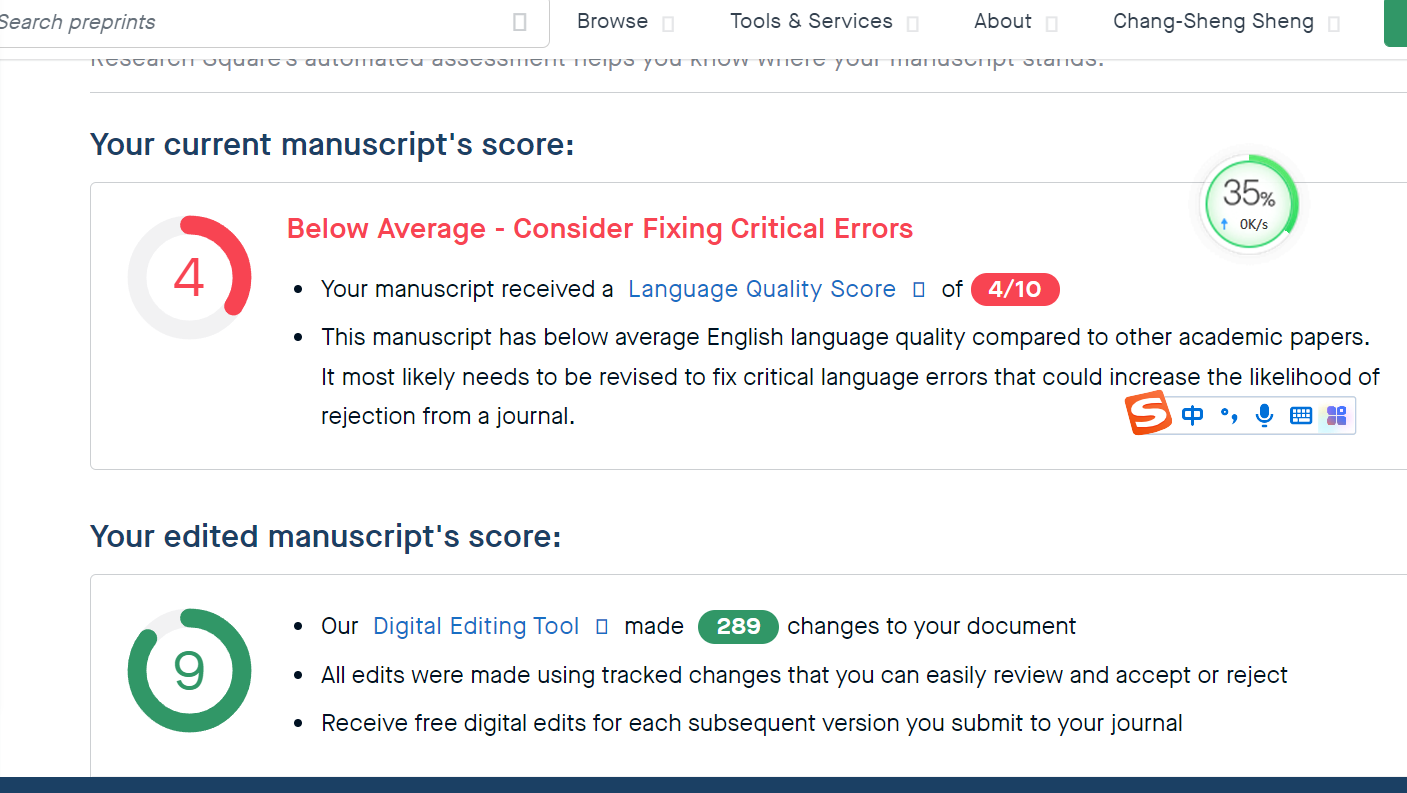

Supplement: Supplementary file 3 — Additional file 3: [file 12944_2022_1628_MOESM3_ESM.png]
