# Supplementary material for: Prognostic significance of visit-to-visit variability, and maximum and minimum LDL cholesterol in diabetes mellitus
Source: Lipids Health Dis. 2022 Feb 10;21:19. doi: 10.1186/s12944-022-01628-8 (PMC8832816; doi:10.1186/s12944-022-01628-8)
Supplement: Supplementary file 4 — Additional file 4: [file 12944_2022_1628_MOESM4_ESM.docx]

**Supplemental files**

**Prognostic significance of visit-to-visit variability, and maximum and minimum LDL cholesterol in diabetes mellitus**

Chang-Sheng Sheng^1*^, Ya Miao^2*^, Lili Ding^3^, Yi Cheng^1^, Dan Wang^1^, Yulin Yang^2^, and Jingyan Tian^2^

^1^Department of Cardiovascular Medicine, State Key Laboratory of Medical Genomics, Shanghai Key Laboratory of Hypertension, Shanghai Institute of Hypertension, Ruijin Hospital, Shanghai Jiao Tong University School of Medicine, Shanghai, China

^2^ State Key Laboratory of Medical Genomics, Clinical Trial Center, Shanghai Institute of Endocrine and Metabolic Diseases, Department of Endocrinology and Metabolism, Ruijin Hospital, Shanghai Jiaotong University School of Medicine, Shanghai, China

^3^Shanghai Key Laboratory of Complex Prescriptions and MOE Key Laboratory for Standardization of Chinese Medicines, Institute of Chinese Materia Medica, Shanghai University of Traditional Chinese Medicine, Shanghai, China.

**Table S1. LDL_c concentration during follow-up**

|  | **Fenofibrate** | | **Placebo** | |
| --- | --- | --- | --- | --- |
|  | **n** | **mean±SD, mg/dl** | **n** | **mean±SD, mg/dl** |
| **Baseline** | 2673 | 100.0±30.3 | 2666 | 101.2±31.0 |
| **The 4^th^ month** | 2597 | 90.3±25.7 | 2602 | 91.5±27.4 |
| **The 8^th^ month** | 2583 | 90.3±26.6 | 2587 | 92.0±27.5 |
| **The 12^th^ month** | 2589 | 89.2±25.5 | 2589 | 90.2±26.4 |
| **The 24^th^ month** | 2504 | 87.2±26.6 | 2483 | 87.7±26.3 |
| **The 36^th^ month** | 2410 | 83.4±26.0 | 2372 | 83.4±27.1 |
| **The 48^th^ month** | 1802 | 83.1±26.1 | 1789 | 81.7±26.6 |

**Table S2. Hazard ratios for 1-SD increase of mean, maximum and minimum LDL cholesterol during follow-up for Outcomes**

| **Correlate (+ 1 SD)** | **Model** |  | **Total population (n=5340)** | |  | **Fenofibrate（n=2673）** | |  | **Placebo (n=2667)** | |
| --- | --- | --- | --- | --- | --- | --- | --- | --- | --- | --- |
|  |  |  | **HR (95%CI)** | ***P*** |  | **HR (95%CI)** | ***P*** |  | **HR (95%CI)** | ***P*** |
| **Primary outcome** |  |  |  |  |  |  |  |  |  |  |
| Mean (+19.8) | None |  | 1.15 (1.06-1.25) | 0.001 |  | 1.11 (0.98-1.26) | 0.09 |  | 1.19 (1.06-1.33) | 0.004 |
|  | Maximum |  | 1.07 (0.93-1.23) | 0.36 |  | 0.94 (0.77-1.15) | 0.54 |  | 1.20(0.99-1.45) | 0.058 |
|  | Minimum |  | 1.26 (1.10-1.44) | 0.001 |  | 1.35 (1.12-1.64) | 0.002 |  | 1.17 (0.97-1.42) | 0.11 |
| Maximum (+29.6) | None |  | 1.15 (1.06-1.25) | 0.0008 |  | 1.17 (1.04-1.32) | 0.01 |  | 1.14 (1.01-1.27) | 0.03 |
|  | Mean |  | 1.10 (0.95-1.26) | 0.20 |  | 1.23 (1.01-1.50) | 0.04 |  | 0.98 (0.81-1.19) | 0.87 |
|  | Minimum |  | 1.15 (1.05-1.26) | 0.003 |  | 1.23 (1.08-1.41) | 0.002 |  | 1.08 (0.95-1.23) | 0.24 |
| Minimum (+17.8) | None |  | 1.07 (0.98-1.17) | 0.12 |  | 0.99 (0.87-1.12) | 0.82 |  | 1.15 (1.03-1.29) | 0.02 |
|  | Mean |  | 0.89 (0.78-1.02) | 0.10 |  | 0.77 (0.64-0.94) | 0.01 |  | 1.02 (0.84-1.23) | 0.88 |
|  | Maximum |  | 1.00 (0.91-1.10) | 0.99 |  | 0.89 (0.77-1.41) | 0.11 |  | 1.11 (0.98-1.26) | 0.12 |
| **Total mortality** |  |  |  |  |  |  |  |  |  |  |
| Mean (+19.8) | None |  | 1.31 (1.19-1.45) | <0.0001 |  | 1.25 (1.07-1.45) | 0.004 |  | 1.36 (1.19-1.55) | <0.0001 |
|  | Maximum |  | 1.73 (1.44-2.06) | <0.0001 |  | 1.35 (1.04-1.77) | 0.03 |  | 2.06 (1.62-2.62) | <0.0001 |
|  | Minimum |  | 0.96 (0.80-1.15) | 0.68 |  | 1.01 (0.78-1.32) | 0.94 |  | 0.91 (0.71-1.17) | 0.47 |
| Maximum (+29.6) | None |  | 1.11 (1.00-1.23) | 0.04 |  | 1.16 (0.99-1.35) | 0.06 |  | 1.08 (0.94-1.24) | 0.30 |
|  | Mean |  | 0.71 (0.59-0.86) | 0.0003 |  | 0.90 (0.69-1.19) | 0.46 |  | 0.58 (0.45-0.76) | <0.0001 |
|  | Minimum |  | 0.91 (0.80-1.03) | 0.13 |  | 1.02 (0.85-1.22) | 0.85 |  | 0.83 (0.70-0.99) | 0.03 |
| Minimum (+17.8) | None |  | 1.42 (1.28-1.56) | <0.0001 |  | 1.31 (1.13-1.53) | 0.0005 |  | 1.50 (1.31-1.70) | <0.0001 |
|  | Mean |  | 1.46 (1.22-1.75) | <0.0001 |  | 1.30 (0.999-1.67) | 0.05 |  | 1.61 (1.26-2.07) | 0.0001 |
|  | Maximum |  | 1.48 (1.32-1.67) | <0.0001 |  | 1.30 (1.09-1.55) | 0.004 |  | 1.64 (1.40-1.91) | <0.0001 |
| **Cardiovascular mortality** |  |  |  |  |  |  |  |  |  |  |
| Mean (+19.8) | None |  | 1.34 (1.16-1.54) | <0.0001 |  | 1.30 (1.05-1.61) | 0.018 |  | 1.36 (1.12-1.63) | 0.001 |
|  | Maximum |  | 1.68 (1.31-2.16) | <0.0001 |  | 1.22 (0.84-1.77) | 0.29 |  | 2.16 (1.53-3.04) | <0.0001 |
|  | Minimum |  | 0.80 (0.61-1.04) | 0.10 |  | 0.93 (0.63-1.37) | 0.70 |  | 0.68 (0.46-0.99) | 0.048 |
| Maximum (+29.6) | None |  | 1.15 (0.99-1.32) | 0.06 |  | 1.26 (1.02-1.56) | 0.03 |  | 1.05 (0.87-1.28) | 0.60 |
|  | Mean |  | 0.75 (0.57-0.97) | 0.03 |  | 1.08 (0.75-1.56) | 0.69 |  | 0.55 (0.38-0.80) | 0.002 |
|  | Minimum |  | 0.89 (0.75-1.06) | 0.19 |  | 1.09 (0.85-1.40) | 0.50 |  | 0.75 (0.59-0.96) | 0.02 |
| Minimum (+17.8) | None |  | 1.54 (1.34-1.77) | <0.0001 |  | 1.43 (1.15-1.78) | 0.001 |  | 1.63 (1.36-1.96) | <0.0001 |
|  | Mean |  | 1.87 (1.43-2.44) | <0.0001 |  | 1.53 (1.03-2.26) | 0.036 |  | 2.24 (1.56-3.23) | <0.0001 |
|  | Maximum |  | 1.64 (1.39-1.93) | <0.0001 |  | 1.37 (1.06-1.77) | 0.015 |  | 1.87 (1.51-2.33) | <0.0001 |

Model indicates which LDL index was entered into the models in addition to the predictor variable per se. None indicates that no LDL cholesterol index was entered in the model. All models were adjusted for mean of lipid during visits, therapy group (if applicable), sex, and baseline age, education, body mass index, systolic and diastolic blood pressure, smoking, drinking, and fasting plasma glucose.
